# Supplementary material for: Probing three-dimensional mesoscopic interfacial structures in a single view using multibeam X-ray coherent surface scattering and holography imaging
Source: Nat Commun. 2023 Sep 18;14:5795. doi: 10.1038/s41467-023-39984-3 (PMC10507109; doi:10.1038/s41467-023-39984-3)
Supplement: Supplementary file 1 — Supplementary Information [file 41467_2023_39984_MOESM1_ESM.pdf]

Supplementary Information

Probing three-dimensional mesoscopic interfacial structures in a single view using multibeam x-ray coherent surface scattering and holography imaging

Miaoqi Chu[1, a], Zhang Jiang[1], Michael Wojcik[1], Tao Sun[1,*], Michael Sprung[2], and Jin Wang[1, b]

[1] *X-ray Science Division, Argonne National Laboratory, Lemont, Illinois 60439, USA*
[2] *Deutsches Elektronen-Synchrotron (DESY), Notkestr. 85, 22607 Hamburg, Germany*
[*] *Present Address: Department of Materials Science and Engineering, University of Virginia, Charlottesville, Virginia 22904, USA*
[a, b] *To whom the correspondence should be addressed: mqichu@anl.gov, wangj@anl.gov*

1 Momentum Transfer in the Grazing Incident Geometry

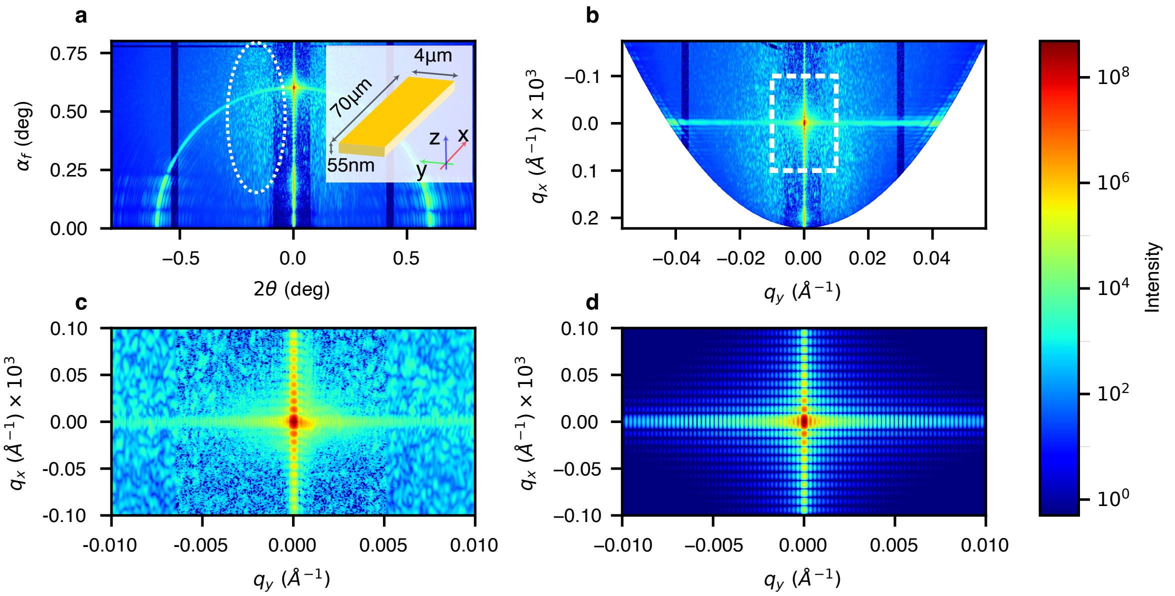

Figure S1: General description of the coherent surface scattering imaging data in the kinematical regime. (a) The raw scattering pattern of the single bar sample described in the main text in the $\alpha_f - 2\theta$ representation. The scattering speckles in the white ellipses originate from the interfacial roughness of the Au sample. The deep blue shadow near $2\theta = 0$ corresponds to the position of the beamstop. When the beamstop is removed to acquire the missing scattering information, the exposure time is shortened to accommodate the detector's dynamic range, resulting in poor photon statistics. (b) The scattering pattern is converted to $q_x - q_y$ coordinates. The region inside the white box corresponds to the low-q_x and -q_y region, where dynamical multiple scattering is less important. This region can be used for kinematic reconstructions. (c) zoomed-in view of the white box in (b), which resembles conventional coherent scattering from a rectangular in-plane pattern. (d) Simulated speckle intensity map from the rectangular in-plane pattern.

In kinematic scattering approximation, the momentum transfers in the grazing incident geometry can be written as

$$q_x = \frac{2\pi}{\lambda}(\cos\alpha_f \cos 2\theta - \cos\alpha_i), \qquad \text{(S1)}$$

$$q_y = \frac{2\pi}{\lambda}\cos\alpha_f \sin 2\theta, \qquad \text{(S2)}$$

$$q_z = \frac{2\pi}{\lambda}(\sin\alpha_i + \sin\alpha_f), \qquad \text{(S3)}$$

where λ is the x-ray wavelength, α_i, α_f, and 2θ are the incident angle, the meridional exit angle, and the azimuthal scattering angle, respectively.

With the conversion equations, the raw scattering patterns in the $\alpha_f - 2\theta$ representation, shown in Fig. S1a, can be remapped to the $q_x - q_y$ plane, as shown in Fig. S1b. Although the q_z component is contained in each detector pixel, the quantity of $\exp(-iq_z L_z)$ varies slowly for the typical planar samples (thickness $L_z \sim 1 - 100$ nm) studied with coherent surface x-ray scattering. Thus the q_z component can usually be excluded when analyzing the in-plane structure. The region far away from the dynamical scattering regime (inside the white box in Fig. S1b), also illustrated in Fig. S1c, can be used to reconstruct the in-plane

structure, provided the over-sampling ratio is sufficiently high. The oscillatory intensity along q_x and q_y directions is the signature of the pattern length (70 μm) along and the width (4 μm) perpendicular to the X-ray beam, respectively. However, during the experiment, due to several issues, The scattering pattern from a rectangular sample of the same dimension (70×4 μm^2, $L_x \times L_y$) in Fig. S1d resembles the scattering pattern shown in Fig. S1c. Examples of successful in-plane reconstruction can be found elsewhere [1, 2]. The mismatch in speckle intensity (experimental data in Fig. S1c vs. simulation in Fig. S1d) can come from three factors: 1) an artifact from the data collection when we used a stripe-shaped attenuator in front of the detector to reduce the intense reflected beam so we could make the exposure time long enough for obtaining a sufficiently high signal-to-noise ratio for the fringes for $\alpha_f < 0.3°$, the focus of the experiment. The shadow of the attenuator is readily observable in all the experimental images; 2) The edge roughness of the sample can reduce the fringe contrast and intensity; 3) The detector with the 75-μm pixel size at the 5-m position just barely resolves the 4-μm bar width. Scattering in the kinematic regime is not the focus of this work, and it will not be discussed in detail elsewhere in this article and the SI document.

We found that the most observable contribution from the surface pattern's interface and edge roughness is in the kinematic scattering regime, as shown in Fig. S1. The dominating effect of interfacial roughness is from the Au pattern surface, where the contrast is the greatest. The scattering is observed in the experiment data (Fig. 1b) and modeled in all simulations throughout the article (see Figs. 2c and 3c, and Fig. S2b). However, the simulation of the surface-roughness scattering is based on the Fourier transform of statistical roughness models. The simulation method is not the focus of this work and will be the subject of our future publications. Therefore, we will not elaborate on the simulation method elsewhere in the article. The signature of edge roughness, on the other hand, is also observable in the higher exit angle α_f area in Fig. S1c (and other experimental scattering patterns) as the lost speckle contrast and fast diminishing scattering intensity in both q_x and q_y directions near the specular reflection. We did not try to simulate the contrast and intensity reduction effects. Still, we speculate that a similar method to model x-ray reflectivity data with the presence of interfacial roughness (as in Ref. 31) can be used for quantifying the statistical distribution (correlation) of the edge roughness. Again, the roughness issues are not closely related to the focus of this manuscript and will be the subject of our future publications on the high-resolution reconstruction of the scattering patterns based on Fourier transform methods.

2 Born Approximation Approach

The kinematic approach or Born approximation (BA) is the most straightforward method for computing the far-field scattering patterns. It assumes that each electron in the path of x-rays receives the unperturbed incoming wave and scatters it as a spherical wave. The far-field scattering pattern is the superposition of all scattered waves, which can be simplified as the modulus squared of the Fourier transform (FT) of the electron density profile ($\rho(\mathbf{r})$), as illustrated in Fig. S2a.

$$I(\mathbf{q}) = \left| \iiint \rho(\mathbf{r}) e^{-i\mathbf{q}\cdot\mathbf{r}} d\mathbf{r} \right|^2 . \tag{S4}$$

BA is widely used in transmission x-ray scattering due to the weak interaction (small scattering cross-section) between x-rays and electrons. The simulation result of the sample scattering based on BA is shown in Fig. S2b, and compared to the experimental data (Fig. S2c). The difference between the BA simulation and the experimental data is immediately apparent, especially at low exit angles ($\alpha_f < 0.3°$).This is because, in the grazing-incidence reflection geometry, the incident and scattered waves can be manipulated significantly by the substrate and the planar sample, thanks to the strong reflection at the surfaces and interfaces. As a result, BA fails to predict the scattering patterns satisfactorily. The simulated scattering pattern lacks all features offered in the experimental data but contains four widely spaced nodes on the arc, which originates from the z component (the bar's thickness) of the pattern's form factor.

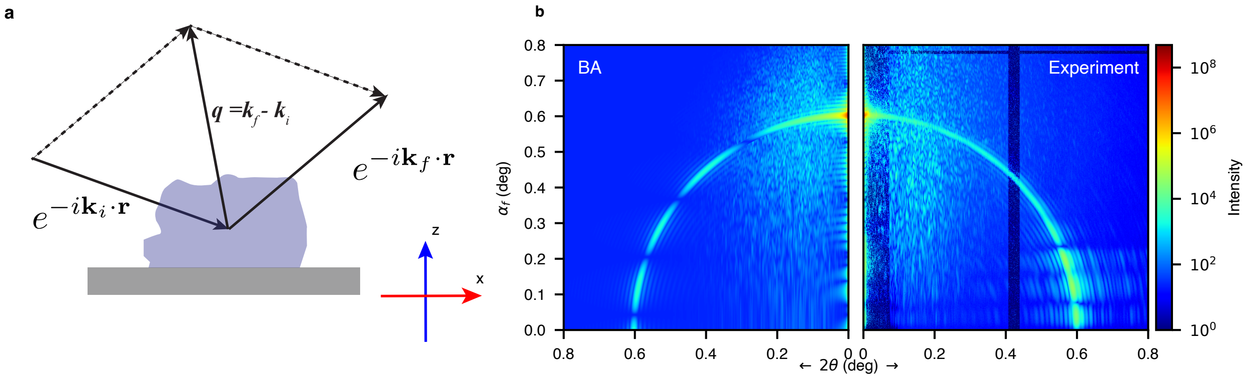

Figure S2: Simulation of surface coherent scattering pattern using existing Born approximation (BA, or kinematic approach). (a) Schematic of BA scattering wave vector transfer in the reflection geometry (not to scale). (b) The scattering pattern (left) was calculated with the BA algorithm as compared to the experimental data (right panel). The mismatch is significant in the scattering images except for the area around $2\theta = 0°$ and $\alpha_f = 0.6°$ where the momentum transfer q_x and q_y are both close to 0 (kinematic regime). Therefore, the BA method is not a viable solution for evaluating the scattering from substrate-supported surface patterns in the reflection geometry other than the kinematic regime.

3 Details about FE-DWBA

We divide the sample and its surroundings into a 3D grid, not only in the out-plane direction (layers in conventional DWBA) but also in the in-plane direction (x-y), as shown in Fig. 3a. In the top view, each box is a stack of layers used in conventional DWBA. The size of the cell is determined by the resolution defined on the detector, $r_t = 2\pi/\max(q_t)$ for t in (x, y, z). This fine grid accounts for the sample's in-plane and out-of-plane 3D inhomogeneity within the coherent volume of the x-ray beam.

The x-ray's propagation can be described as a scalar wave with the Helmholtz equation,

$$(\nabla^2 + k_0^2)\psi = 4\pi r_e \rho(\mathbf{r})\psi, \tag{S5}$$

in which k_0 is the wave vector in vacuum, r_e is the classic electron radius, while $\rho(\mathbf{r})$ is the complex electron density.

According to Snell's law, the refracted angle α_p in the p_{th} layer with refractive index of n_p is,

$$\cos\alpha_i = n_p \cos\alpha_p. \tag{S6}$$

Thus, the perpendicular and parallel components of the wave vector can be written as,

$$k^i_{p,z} = n_p k_0 \sin\alpha_p = k_0(n_p^2 - \cos^2\alpha_i)^{1/2}, \tag{S7}$$

$$k^i_{p,xy} = n_p k_0 \cos\alpha_p \approx k_0, \tag{S8}$$

in which k_0 is the wave number in vacuum, $k_0 = 2\pi/\lambda$.

The eigenstate of the unperturbed incident beam, which is determined by α_i can be written as,

$$\psi^i_p = T^i_p e^{i\mathbf{k}^i_p\cdot\mathbf{r}} + R^i_p e^{i\mathbf{k}'^i_p\cdot\mathbf{r}} = (T^i_p e^{ik^i_{p,z}r_z} + R^i_p e^{-ik^i_{p,z}r_z})e^{i\mathbf{k}^i_0\cdot\mathbf{r}_{p,\parallel}}, \tag{S9}$$

in which $p = (x_p, y_p, z_p)$ is a three-element tuple to index the cell's position in the 3D grid. $\mathbf{k}^i_p$ and $\mathbf{k}'^i_p$ are two mirrored wave vectors with respect to the surface normal. r_z and $\mathbf{r}_{p,\parallel}$ are the perpendicular and parallel component position of the p_{th} cell. T^i_p and R^i_p are the transmission and reflection in the p_{th} cell, obtained by the Fresnel equation.

The exit eigenstate which is determined by $(2\theta, \alpha_f)$ on the detector in the p_{th} cell, is the time-reversed state of the incident beam with a wave vector of $-\mathbf{k}_p^f$.

$$\psi_p^f = (T_p^f e^{-i\mathbf{k}_p^f\cdot\mathbf{r}} + R_p^f e^{-i\mathbf{k}_p'^f\cdot\mathbf{r}})^* = \left[(T_p^f e^{-ik_{p,z}^f z} + R_p^f e^{ik_{p,z}^f z})e^{-i\mathbf{k}_0^f\cdot\mathbf{r}_{p,\parallel}}\right]^*. \tag{S10}$$

The scattering from the grid is the sum of all scattering terms in phase,

$$I \propto \left|\sum_{x_p,y_p}\sum_{z_p}\langle\psi_p^f|\rho_p|\psi_p^i\rangle + \sum_{x_e,y_e\in\text{boundaries}}\sum_{z_e}\langle\psi_{e_1}^f|\rho_e|\psi_{e_2}^i\rangle\right|^2, \tag{S11}$$

in which ρ_p and ρ_e are the electron density in cell p and e. The first term in the modulus-squared operator is the scattering for a single cell. The second term is the scattering from the cells with different waves, i.e, the cells sitting on the boundaries.

To simplify the computing, we first sum all cells with the same x and y index in the z-direction. Here the summation is done for the second term as the result for the first term can be easily obtained by substitution.

$$\begin{aligned}\sum_{z_e=1}^{N}\langle\psi_{e_1}^f|\rho_e|\psi_{e_2}^i\rangle &= \sum_{z_e=1}^{N}(T_{e_1}^f e^{-i\mathbf{k}_{e_1}^f\cdot\mathbf{r}} + R_{e_1}^f e^{-i\mathbf{k}_{e1}'^f\cdot\mathbf{r}})(T_{e_2}^i e^{i\mathbf{k}_{e_2}^i\cdot\mathbf{r}} + R_{e_2}^i e^{i\mathbf{k}_{e_2}'^i\cdot\mathbf{r}})\rho_e \\ &= \sum_{z_e=1}^{N}(T_{e_1}^f e^{-ik_{e_1,z}^f z} + R_{e_1}^f e^{ik_{e_1,z}^f z})(T_{e_2}^i e^{ik_{e_2,z}^i z} + R_{e_2}^i e^{-ik_{e_2,z}^i z})e^{-i(\mathbf{k}_{e_1}^f-\mathbf{k}_{e_2}^i)_{xy}\cdot\mathbf{r_{xy}}}\rho_e \\ &= \rho_e'(e_1,e_2,i,f)e^{-i\mathbf{q}_{xy}\cdot\mathbf{r}_{xy}},\end{aligned} \tag{S12}$$

in which $\mathbf{q}_{xy} = (\mathbf{k}_{e_1}^f - \mathbf{k}_{e_2}^i)_{xy}$ is the in-plane component of wave vector transfer, ρ_e' is the effective electron density due to the distorted wave (dynamical scattering) effect on the surface which is a function of the initial and final states (annotated as i and f, which are determined by α_i, α_f and 2θ),

$$\rho_e'(e_1,e_2,i,f) = \rho_e\sum_{z_e=1}^{N}\sum_{m=1}^{4}D_m^{1,2}e^{-iq_{z,4}^{1,2}z}, \tag{S13}$$

$$D_1^{1,2} = T_{e_1}^f T_{e_2}^i \qquad q_{z,1}^{1,2} = k_{e_1,z}^f - k_{e_2,z}^i, \tag{S14}$$

$$D_2^{1,2} = R_{e_1}^f T_{e_2}^i \qquad q_{z,2}^{1,2} = -k_{e_1,z}^f - k_{e_2,z}^i, \tag{S15}$$

$$D_3^{1,2} = T_{e_1}^f R_{e_2}^i \qquad q_{z,3}^{1,2} = k_{e_1,z}^f + k_{e_2,z}^i, \tag{S16}$$

$$D_4^{1,2} = R_{e_1}^f R_{e_2}^i \qquad q_{z,4}^{1,2} = -k_{e_1,z}^f + k_{e_2,z}^i. \tag{S17}$$

The four terms in Equation S13 correspond to four possible scattering paths on the surface.

For planar objects studied with grazing incident x-ray scattering, many of the cell stacks (stack of layers with the same x and y index) can share the same vertical electron density profile in the z direction, just like a "grain". They can be grouped together to $G_j = \{g_i^1, g_i^2, \cdots, g_i^{N_j}\}$, in which each element g_i that share the same profile. The whole sample can have multiple grains, $G = \{G_j|j = (1,2,3,...)\}$. Similarly, the cells on edge (boundaries between different grains) can also be grouped according to their surroundings cells (e_1 and e_2), and their vertical electron density profile. All possible edge group can be written as set E, which consist individual group of $E_j = \{e_i^1, e_i^2, \cdots, e_i^{N_j}\}, j = (1,2,3,...)$. Equation S11 can be simplified to,

$$\begin{aligned} I &\propto \left|\sum_{G_j\in G}\sum_{g\in G_j}\rho_g'(g,g,i,f)e^{-i\mathbf{q}_{xy}\cdot\mathbf{r_{xy}}} + \sum_{E_j\in E}\sum_{e\in E_j}\rho_e'(e_1,e_2,i,f)e^{-i\mathbf{q}_{xy}\cdot\mathbf{r_{xy}}}\right|^2 \qquad \text{(S18)} \\ &= \left|\text{FT}_2\{\rho_g'(x,y)\} + \text{FT}_2\{\rho_b'(x,y)\}\right|^2, \qquad \text{(S19)}\end{aligned}$$

in which FT_2 is the Fourier transform in 2 dimension, $\rho'_g(x, y)$ is the effective electron density for single cells and $\rho'_e(x, y)$ is the effective electron density profile on the boundaries,

$$\begin{aligned} \rho'_g(x, y) &= \sum_{G_j \in G} \sum_{g \in G_j} \rho'_g(g, g, i, j)\delta((x, y), g), && \text{(S20)} \\ \rho'_b(x, y) &= \sum_{E_j \in E} \sum_{g \in E_j} \rho'_e(e_1, e_2, i, j)\delta((x, y), e), && \text{(S21)} \end{aligned}$$

in which δ is the Kronecker delta function.

In the modulus-squared operator of Equation S19, the first term describes the scattering wave from each cell, while the second term describes the scattering on the edge cells (between two grains). Note that both terms account for the dynamical scattering effects due to the use of the effective electron density profile.

In the case of partial coherent x-ray scattering, the probe can have multiple modes, each with its own wavefront; the scattering intensity can be written as,

$$I \propto \sum_k w_k \left| \mathrm{FT}_2\{[\rho'_g(x, y) + \rho'_b(x, y)]P_k(x, y)\} \right|^2, \tag{S22}$$

in which w_k is the weight for the k_{th} mode and $P_k(x, y)$ is the complex wavefront projected on the sample.

4 Scattering patterns collected at different incident angles.

To understand the x-ray version of Lloyd's mirror concept, with the single-bar sample, we recorded surface scattering images at several different grazing-incidence angles. Only the images taken at (a) 0.4°, (b) 0.6°, and (c) 0.8° are shown in Fig. S3. It is immediately apparent that the overall scattering patterns change dramatically as the incident and exit angle increase. In the meantime, the interference modulations at the low exit angles (below 0.4°) remain invariant. Therefore, the interference is not sensitive to the incident angle but hinged on the sample morphology. The coherent scattering from multiple views may provide enough oversampling for the reconstruction of the surface structure, which will not be discussed further in this article. Rather, we will discuss the feasibility and reconstruction algorithms in our future work.

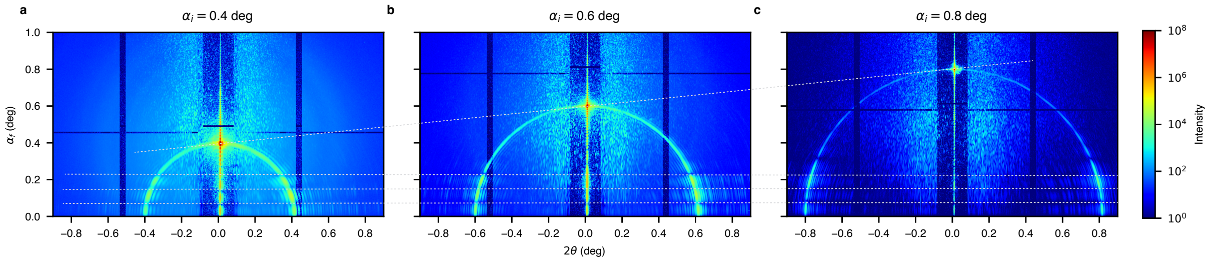

Figure S3: Coherent surface scattering patterns of the bar sample at different incident angles 0.4°, 0.6°, and 0.8°. The inclined broken line shows the linear increase of the reflection angles of the same values as the incident angles. The four equally spaced parallel broken lines below 0.4° exit angle indicate that period of fringes along the α_f axis is independent of the incident angle.

5 Details on the first principles approach

FE-DWBA, despite the effort such as grouping similar cells and using GPU parallelization, is still computation-intense. Here we introduce more assumptions to further simplify the FE-DWBA approach.

- The signal collected in CSSI and GISAXS experiments at high α_i and low α_f regions are particularly important thanks to its high signal-to-noise ratio and separation of the specular signal. For these regions, the first two terms in Equation S13 dominate because the reflectance of the incident beam R^i is low. Instead of incorporating the dynamical scattering effect into the effective electron density (Equation S13), we consider the final scattering as the superposition of scattering from the sample, its edge, and the mirrored image of its edge after reflection on the substrate.
- For the samples with high-z materials which have high critical angles, x-ray at a low grazing incident angle can only penetrate a limited depth with respect to the top surface of the sample. In other words, x-rays cannot 'see' the interface between the sample and the substrate. The scattering in the 1st term of Equation S19 can be approximated as a Fourier transform of the region's in-plane shape.

With these assumptions, the scattering intensity in Equation S19 can be simplified to,

$$I(\alpha_i, \alpha_f, 2\theta) \propto \left|\mathrm{FT}_2\{\rho_g(x, y)\} + \mathrm{FT}_2\{\rho_b(x, y)(e^{-iq_z h} + e^{iq_z h})R(\alpha_f)\}\right|^2, \tag{S23}$$

in which h is the height of cells on the boundaries, $R(\alpha_f)$ is the reflection at the exit angle of α_f which is also a function of q_z. $R(\alpha_f)$ modulates the reflected wave both in amplitude and phase. The phase change due to reflection is α_f at 0° exit angle and gradually changes to 0 at the critical angle.

The wave transfer vector defined by the detector in the GISAXS/CSSI geometry is not on a cartesian grid. Usually, this can be best solved using a non-uniform Fourier transform (NUFT). If the form factor of the sample is known to have a closed form, it can significantly accelerate the forward scattering simulation.

6 Origin of the two arcs

We observed two arcs in the scattering pattern of the double-bar sample (Fig. 4c) which originates from the misalignment between the bottom bar and the top bar during sample preparation. Both bars, under a coherent x-ray beam, will create an arc-shaped scattering pattern on the detector, which is essentially the $q_x = 0$ curve as shown in Fig. S4a. When the two bars are well aligned, the scattering wave from the two bars will add together in phase, resulting in a single arc shown in Fig. S4b.

From the quantitative modeling in the main text, we found that the misalignment angle is about $\gamma = 0.009°$ counterclockwise, which is possible during the sample preparation stage (Note the top bar is fabricated on the top of the bottom bar, which requires the alignment step using SEM.)

7 Scattering patterns of the stacked bar at different base heights

Here we simulate the scattering pattern of the stacked-bar sample at three different bottom bar heights (h_b), 55, 65, and 75 nm, while the rest parameters are fixed, as shown in the schematic in Fig. S5a. The scattering patterns are shown in Figs. S5.b-d. The line cuts of the out-arc and inner-arc (both defined in the main text) in Figs. S5.e-f shows the speckle size and phase along the arcs strongly dependent on h_b.

References

[S1] S Roy, D Parks, KA Seu, Run Su, JJ Turner, Weilun Chao, EH Anderson, Stefano Cabrini, and SD Kevan. Lensless x-ray imaging in reflection geometry. *Nature Photonics*, 5(4):243–245, 2011.

[S2] Tao Sun, Zhang Jiang, Joseph Strzalka, Leonidas Ocola, and Jin Wang. Three-dimensional coherent x-ray surface scattering imaging near total external reflection. *Nature Photonics*, 6(9):586–590, 2012.

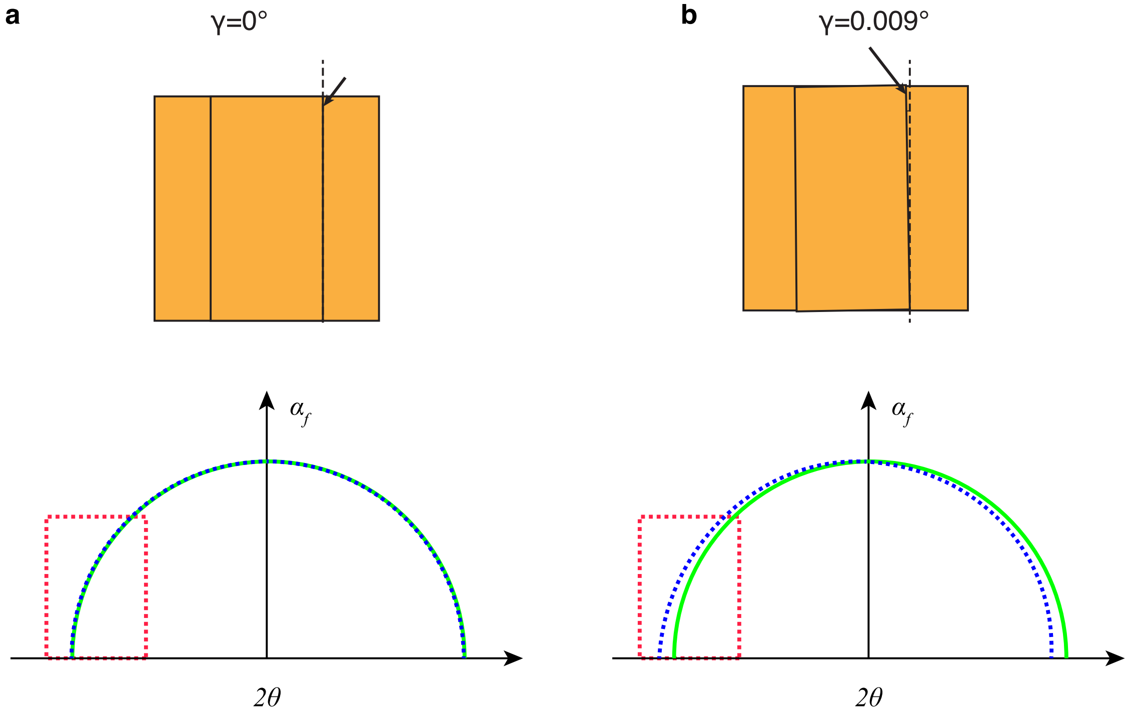

Figure S4: The origin of the double arcs in the scattering pattern of the double bar sample. The bottom bar and the top bar both scatter the x-ray forming an arc-shaped pattern on the detector, shown as the green solid line and blue dashed line. (a) the two arcs will be superimposed in the same position when the two bars are well aligned ($\gamma = 0$). (b). the arc from the top bar (blue-dashed line) is rotated with respect to the arc pattern from the button bar when there is a small angle. The red rectangular marks the region that is analyzed in the main text.

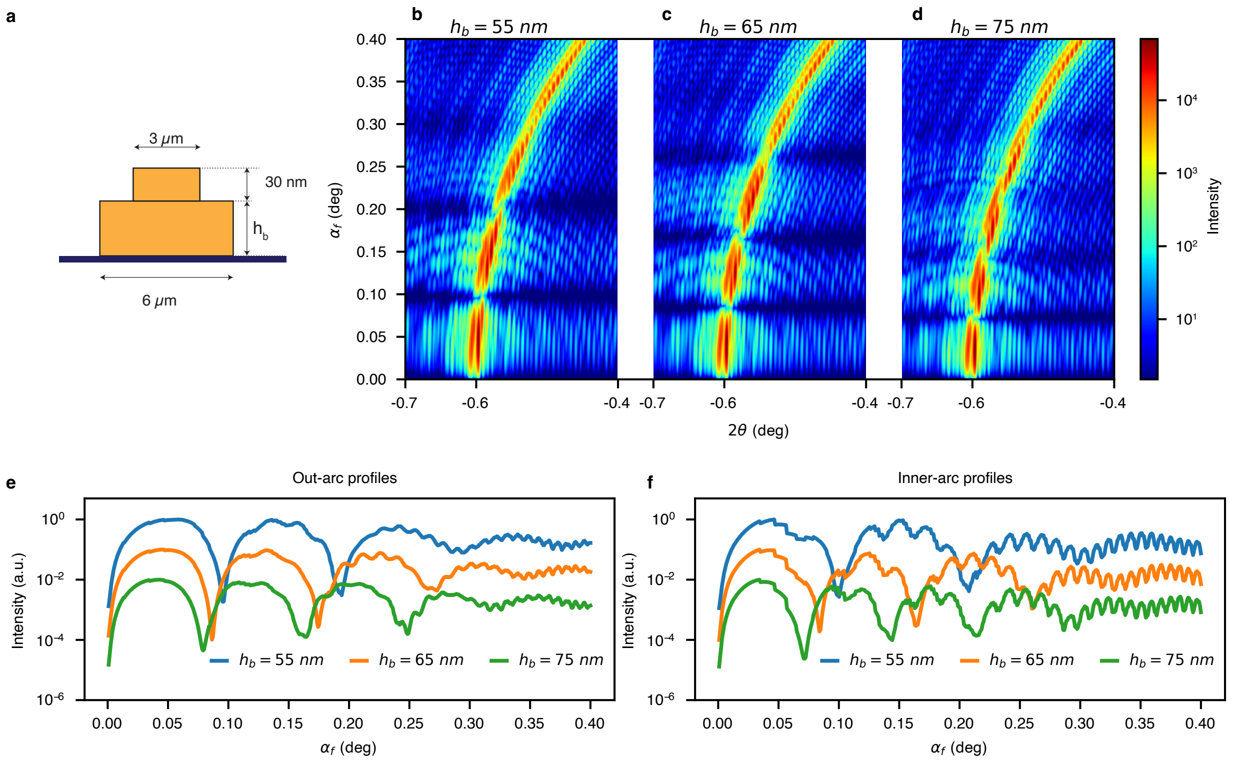

Figure S5: Grazing incidence holography of a stacked-bar pattern with different bottom bar heights. (a) Schematic of the stacked-bar sample pattern with a varying bottom-bar height of h_b but fixed value for the rest parameters. (b)-(d) the scattering patterns for $h_b = 55, 65, 75$ nm respectively. (e)-(f) Line-cuts of the simulated scattering intensity profiles along the out-arc and inner-arc as a function of α_f from 0 to 0.4° as h_b varies. Both line cuts are highly sensitive to h_b.
